# Cell targeting by the bicomponent leukocidin subunit HlgB drives *Staphylococcus aureus* pathophysiology

**DOI:** 10.1016/j.jbc.2025.110592

**Published:** 2025-08-12

**Authors:** Julia Sproch, Rachel Prescott, Hee Jin Kim, Chrispin Chaguza, Sandra Gonzalez, Juliana K. Ilmain, Bo Shopsin, Adam J. Ratner, Victor J. Torres

**Affiliations:** 1 Department of Microbiology, New York University Grossman School of Medicine, New York, New York, USA; 2 Department of Host-Microbe Interactions, St Jude Children’s Research Hospital, Memphis, Tennessee, USA; 3 Center for Infectious Diseases Research, St Jude Children’s Research Hospital, Memphis, Tennessee, USA; 4 Department of Medicine, Division of Infectious Diseases, NYU Grossman School of Medicine, New York, New York, USA; 5 Department of Pediatrics, New York University Grossman School of Medicine, New York, New York, USA

**Keywords:** bacterial pathogenesis, bacterial toxin, erythrocyte, neutrophil, *Staphylococcus aureus*

## Abstract

*Staphylococcus aureus* is a global health concern, resulting in significant disease burden in both hospital and community settings. To establish infection, the bacteria must contend with a multitude of host defense mechanisms, including “nutritional immunity,” in which nutrients are sequestered away from invading pathogens. Importantly, *S. aureus* requires iron for growth during infection, which it acquires through the lysis of erythrocytes (hemolysis). HlgAB, a secreted bi-component pore-forming toxin, contributes to the ability of *S. aureus* to lyse erythrocytes to release heme iron. HlgAB consists of two subunits, the S-subunit HlgA and the F-subunit HlgB. Prior work has shown that the hemolytic activity of HlgAB is dependent on the binding of HlgA to the host receptor Duffy Antigen Receptor for Chemokines (DARC). Here we show that HlgB binds the surface of erythrocytes independent of DARC or HlgA. Our comparative genomic analysis reveals high conservation of *hlgA* and *hlgB* genes across *S. aureus* lineages. By performing structure–function studies, we identified a series of loops within the rim domain of HlgB that are required for the binding of HlgB to erythrocytes and erythrocyte lysis by HlgAB. The importance of HlgB-mediated host targeting was validated in a tissue culture model of *S. aureus*-mediated lysis of primary human erythrocytes, in an *in vivo* murine model of intoxication, and during *in vivo* systemic infection. Altogether, these findings expand our mechanistic insights into how *S. aureus* overcomes nutritional immunity and the role of HlgB in *S. aureus* pathophysiology.

*Staphylococcus aureus* is a causative agent of significant disease worldwide (1, 2, 3, 4). To persist in the harsh environments of the human host, *S. aureus* employs a variety of virulence factors, including bi-component pore forming toxins known as leukocidins (1, 2, 5). *S. aureus* isolates associated with human infection can secrete up to five different leukocidins: LukSF-PV (or PVL), LukED, LukAB (also known as LukGH), HlgAB, and HlgCB. Each leukocidin pair is made of two subunits, an S-subunit that targets host receptors and an F-subunit that binds the S-subunit to form octameric pores (6, 7, 8, 9, 10, 11). Leukocidins tamper with the host immune response by targeting phagocytes, release nutrients by lysing erythrocytes, and promote tissue damage and dissemination by disrupting the endothelium (5, 12).

Notably, differential receptor targeting allows for cell type specificity of the leukocidins (13, 14, 15, 16, 17, 18, 19). Despite this, amino acid alignments of the S-subunits (HlgA, LukE, and LukS-PV) show a high level of sequence identity (66–70%), and the F-subunits (HlgB, LukD, and LukF-PV) have an even higher degree of identity (71–82%) (5). This sequence conservation is likely the reason for the formation of both cognate (*e.g.* HlgA/HlgB) and noncognate (*e.g.* HlgA/LukD) combinations of leukocidin subunits (20). Each toxin subunit is comprised of cap, stem, and rim domains (6, 21, 22). Receptor and cell type tropism is mainly determined by divergence in the sequences of the rim domains (14, 23, 24, 25, 26).

The hypothesized mechanism of leukocidin-mediated lysis is as follows: the S-subunit targets a host cell receptor and recruits the F-subunit for dimerization, oligomerization, and pore formation (27). Pore formation results in loss of membrane integrity and subsequent osmotic lysis of host cells (2). Recently, however, the F-subunit LukF-PV was found to target CD45, an interaction vital for LukSF-PV toxicity (28). Further evidence suggests the contribution of the F-subunit of LukPQ, LukQ, to cellular tropism toward bovine and equine leukocytes (29). Finally, HlgB and LukD have been shown to bind host cells independent of HlgA or LukE (20, 23, 30, 31, 32). Collectively, these findings suggest significance of cell targeting by not only the S-subunits, but the F-subunits as well.

Among the bi-component leukocidins, HlgAB and LukED target and lyse erythrocytes, resulting in the release of hemoglobin (13, 23, 33, 34, 35, 36). The most abundant source of iron in the human body is bound to heme in the form of hemoglobin, which is sequestered inside erythrocytes (13, 37). Heme iron is the preferred iron source for *S. aureus*, and *S. aureus* encodes the iron-determinant system (Isd) to use hemoglobin during infection (37, 38, 39, 40). While much is known about the intricacies of heme iron usage, the molecular mechanism by which *S. aureus* lyses erythrocytes to access intracellular hemoglobin is poorly understood.

HlgAB and LukED lyse erythrocytes by targeting the host receptor Duffy Antigen Receptor for Chemokines (DARC), also known as the atypical chemokine receptor 1 (ACKR1), through direct binding by the S-subunits, HlgA and LukE (13). While both leukocidins are hemolytic to human erythrocytes, HlgAB alone is sufficient in tissue culture models when *S. aureus* is incubated with erythrocytes (13). The importance of HlgAB for *S. aureus* pathobiology is supported by the finding that the *hlgACB* operon is present in over 99% of all sequenced *S. aureus* strains associated with human infection (41). By targeting DARC, HlgAB and LukED also lyse endothelial cells, resulting in vascular disfunction and rapid lethality *in vivo* (12).

Here we dissect the molecular mechanisms of HlgAB-mediated host targeting. We reinforce prior findings that erythrocyte targeting and hemolysis by HlgAB not only involves HlgA, but also requires HlgB (23, 26, 30, 31). In addition, we demonstrate the high conservation of HlgA and HlgB proteins through a comparative genomic analysis. By performing structure–function studies using HlgB and LukF-PV, we show that multiple loops within the HlgB rim domain are required for erythrocyte targeting and lysis. Detailed amino acid swapping studies confirm tyrosine 71 to be essential for erythrocyte binding by HlgB, and erythrocyte lysis by HlgAB (31). Finally, we establish the *in vivo* relevance of HlgB-mediated host targeting using both a murine intoxication model and a murine model of systemic infection.

## Results

### Cell targeting by HlgB is required for HlgAB-mediated hemolysis and murine challenge

**Figure 1:**
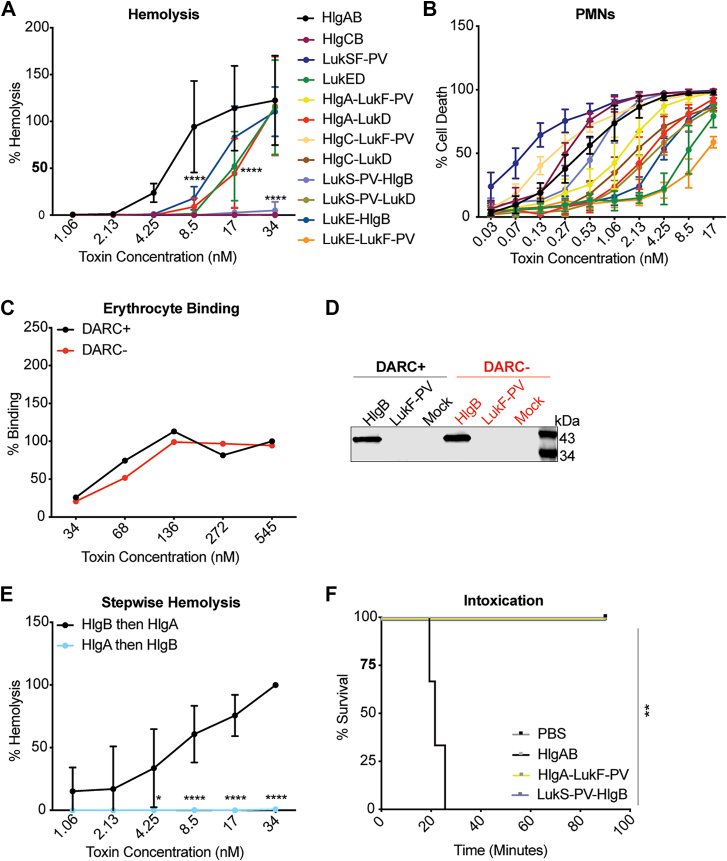
**HlgB is required for HlgAB mediated hemolysis and *in vivo* toxicity.***A*, hemolysis of primary human erythrocytes treated with cognate and noncognate combinations of purified *S. aureus* leukocidin subunits, normalized to Triton-X100-mediated lysis at 100% (n = 4 donors). Of note, the HlgCB, LukSF-PV, HlgA/LukF-PV, HlgC/LukF-PV, HlgC/LukD, LukS-PV/HlgB, LukS-PV/LukD, and LukE/LukF-PV combinations are superimposed in the graph due to a lack of hemolytic activity. *B*, viability of primary human PMNs treated with cognate and noncognate combinations of purified *S. aureus* leukocidin subunits. Normalized to 17 nM HlgAB at 100% (n = 6). *C*, binding of HlgB to primary human erythrocytes, measured by flow cytometry and normalized to 545 nM HlgB binding to DARC + at 100% (one human donor). *D*, binding of HlgB, LukF-PV, or mock control to primary human erythrocytes, measured by immunoblot with anti-His primary antibody (same donors as in Panel C). *E*, stepwise hemolysis of primary human erythrocytes treated with one subunit at a time, washed, then incubated with another subunit. Normalized to 34 nM “HlgB then HlgA” at 100% (n = 4). *F*, survival curve of mice challenged IV with the indicated purified leukocidin subunits (n = 3 mice/group). The data are pooled from at least two independent experiments (except *C*, *D*, and *F*) and represent means ± SD. Of note, HlgA-LukF-PV and LukS-PV-HlgB are superimposed due to a lack of cytotoxic activity *in vivo*. ∗∗∗∗, *p* < 0.0001; ∗∗∗, *p* < 0.001; ∗∗, *p* < 0.01 ∗, *p* < 0.05; *A*, *B*) two-way ANOVA with Sidak’s correction for multiple comparisons; *E*) Mantel-Cox test.

HlgAB and LukED are hemolytic, while LukSF-PV and HlgCB are not (Fig. 1*A* and (13)). The ability of leukocidins to target cells has been previously attributed to S-subunits binding to host receptors (27). However, when creating non-cognate combinations, we found that only combinations with both an S and F subunit from a hemolytic pair (HlgA/LukD and LukE/HlgB) exhibit hemolytic activity (Fig. 1*A*). These data support the notion that both the S and F subunits are involved in erythrocyte lysis. Conversely, all toxin combinations demonstrated lytic ability against primary human polymorphonuclear leukocytes, also known as human neutrophils (hereafter hPMNs) (Fig. 1*B*). HlgA binds erythrocytes *via* DARC (13), so we next assessed the ability of HlgB to bind erythrocytes independently of HlgA or DARC. HlgB bound both DARC-positive and DARC-negative primary human erythrocytes (Figs. 1, *C*, *D* and S1). Because HlgB-bound erythrocytes without HlgA or DARC, we next performed a stepwise hemolysis experiment in which we added one subunit at a time (HlgA or HlgB), incubated, washed, and then added the other. We observed significantly higher levels of hemolysis when cells were first exposed to HlgB followed by HlgA (Fig. 1*E*), supporting the importance of HlgB-mediated host targeting to the hemolysis process.

Next, we investigated the role of HlgB *in vivo* using an established mouse intoxication model (12, 23). When HlgAB is injected intravenously, the toxin results in rapid morbidity due to derangements in the distribution of vascular fluids by targeting endothelial DARC (12). Because HlgA targets DARC on both erythrocytes and endothelial cells, we hypothesize that in addition to erythrocyte targeting, HlgB is also important in endothelial targeting and therefore the *in vivo* intoxication process. Mice were challenged retro-orbitally with cognate and noncognate combinations of leukocidin subunits. While mice intoxicated with HlgAB deteriorated rapidly, those intoxicated with HlgA/LukF-PV or LukS-PV/HlgB fared much better (Fig. 1*F*), suggesting the importance of both HlgA and HlgB *in vivo*. Thus, HlgB is not just required for HlgAB hemolysis, but it is also required for HlgAB toxicity *in vivo.*

### The HlgA and HlgB proteins are highly conserved across *S. aureus* lineages

**Figure 2:**
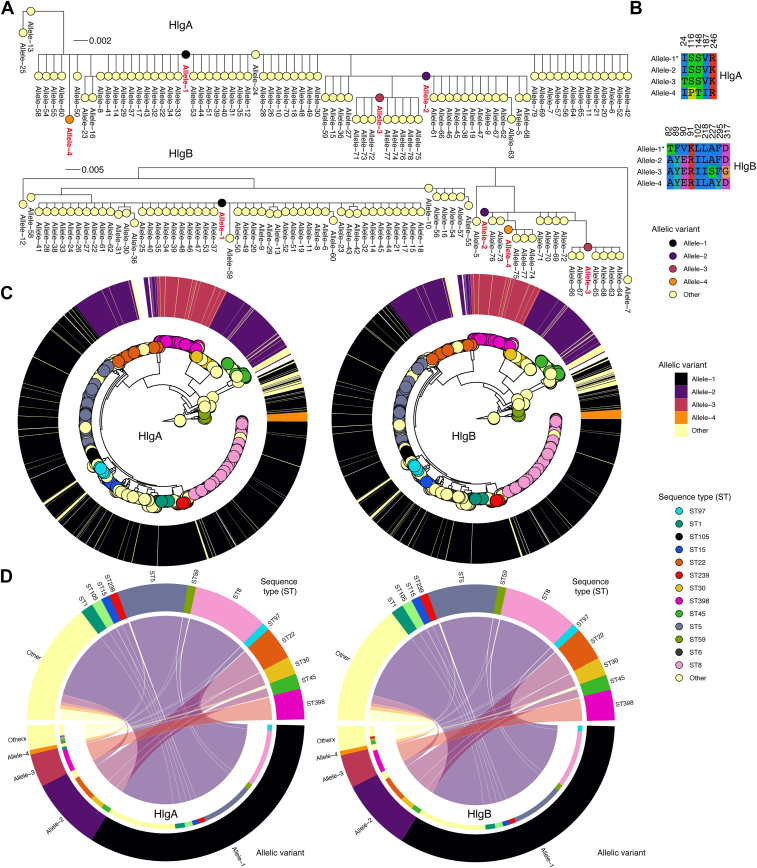
**Conservation and population-level diversity of the HlgA and HlgB proteins.***A*, maximum likelihood phylogeny of the unique HlgA and HlgB allele sequences. The *top* four most frequent alleles of each protein, found in >1% of the genomes, are colored in *red*. *B*, multiple sequence alignment of the top four most frequent alleles showing only positions containing variable amino acids. *C*, a maximum likelihood whole-genome phylogeny showing genetic relatedness of randomly sampled *S. aureus* genomes obtained from GenBank. The tips of the phylogenies are colored based on the sequence types (STs) of the strains while the circular ring surrounding the phylogeny depicts the distribution of different alleles of each protein. *D*, chord diagrams showing the association of different alleles and STs.

To investigate which residues may be important for the activity of these toxins, we conducted a comparative genomic analysis and explored the genetic diversity and sequence conservation of the HlgA and HlgB proteins. First, we assessed the population-level distribution of the two genes across randomly selected *S. aureus* genomes from public nucleotide databases (Tables S1 and S2). We found near-universal presence of the HlgA and HlgB proteins in the genomes highlighting them as part of the core virulence factors of *S. aureus*. Second, we assessed the frequency of different allelic variants of the proteins. We found 79 and 77 unique amino acid allelic variants for HlgA and HlgB, respectively (Fig. 2*A*). We found four alleles in each gene present in >90% of the genomes. The frequencies of these top four most frequent HlgA alleles were 68% (Allele 1), 17% (Allele 2), 7.9% (Allele 3), and 1.1% (Allele 4), and 67% (Allele 1), 14% (Allele 2), 9.4% (Allele 3), and 4% (Allele 4) for HlgB (Figs. 2*B*, S3 and S4). After comparing representative allele sequences, we found a mean number of amino acid differences of 0.15 (range: 0–2) for HlgA and 0.60 (range: 0–3) for HlgB. We found a similar Simpson diversity index of 0.50 for HlgA and HlgB alleles, reflecting limited allelic diversity of both proteins. These findings suggest that most of the *S. aureus* strains express the same HlgA and HlgB allele. For both proteins, notable sequence types (STs) expressing Allele 1 included ST5 and ST8, while ST22 and ST398 expressed Allele 2 and Allele 3, respectively (Fig. 2, *C* and *D*). Altogether, these findings demonstrate high species-wide conservation and minimal genetic diversity of the HlgA and HlgB proteins across the *S. aureus* population.

### Identification of critical regions of HlgB for hemolysis

**Figure 3:**
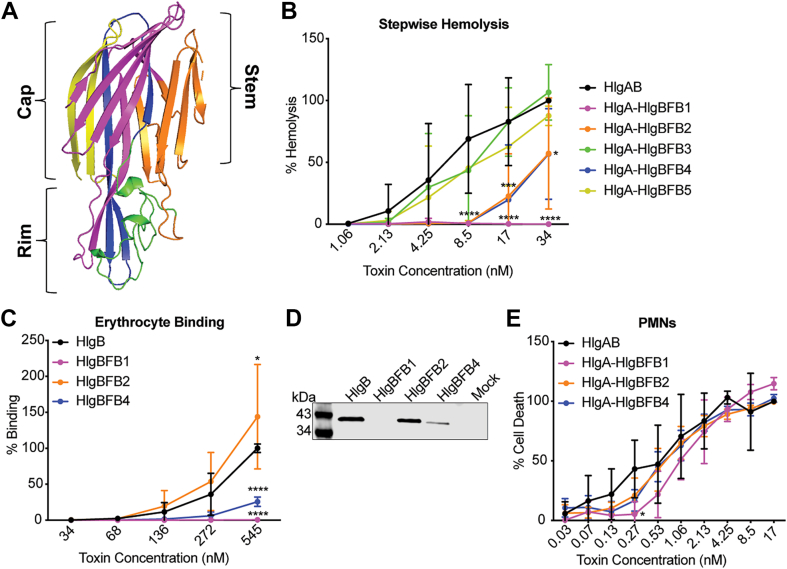
**HlgB domain 1 is necessary for erythrocyte binding by HlgB and lysis by HlgAB.***A*, atomic resolution structure for HlgB (1LKF), with colors indicating 5 domains (Domain 1 is *pink*, Domain 2 is *orange*, Domain 3 is *green*, Domain 4 is *blue*, and Domain 5 is *yellow*). *B*, stepwise hemolysis of primary human erythrocytes treated with WT HlgB or HlgB-LukF-PV chimera, washed, then treated with HlgA. HlgBFB1 indicates HlgB with domain 1 from LukF-PV, HlgBFB2 indicates HlgB with domain 2 from LukF-PV, *etc.* HlgAB at 34 nM was set at 100% (n = 6). *C*, binding of HlgB or HlgB chimeras to primary human erythrocytes as measured by flow cytometry. Data were normalized to 545 nM HlgB binding at 100% (n = 4). *D*, binding of HlgB, chimera, or mock control to primary human erythrocytes, measured by immunoblot with anti-His primary antibody. Immunoblot depicts a representative sample. *E*, viability of primary human PMNs treated with WT HlgAB or the HlgA/HlgB chimeras. Normalized to 17 nM HlgAB at 100% (n = 4–8). The data are pooled from at least two independent experiments and represent means ± SD. ∗∗∗∗, *p* < 0.0001; ∗∗∗, *p* < 0.001; ∗∗, *p* < 0.01 ∗, *p* < 0.05; B-D) two-way ANOVA with Sidak’s correction for multiple comparisons.

Because HlgB exhibits minimal genetic diversity, we decided to compare its amino acid sequence to that of LukF-PV. Although amino acid alignments of HlgB and LukF-PV from CA-MRSA USA300 strain FPR3757 show 71% residue identity, toxin combinations with LukF-PV do not lyse erythrocytes, and LukF-PV does not bind erythrocytes (Fig. 1, *A* and *C*) (5). To identify regions of HlgB important for erythrocyte binding and lysis, we took advantage of the sequence and structure similarity between HlgB and LukF-PV (42, 43). We created chimeras by dividing both proteins into 5 domains and swapping analogous regions of LukF-PV into HlgB one by one (Fig. 3*A*). The chimeric proteins were purified and then tested for activity using the stepwise hemolysis assays in which the F-subunits (HlgB or chimera) were added first, followed by HlgA. HlgA/HlgBFB3 (residues 178–222) and HlgA/HlgBFB5 (residues 268–299/301) showed similar levels of hemolysis to WT HlgAB, HlgA/HlgBFB2 (residues 98–177) and HlgA/HlgBFB4 (residues 223–267) showed intermediate levels of hemolysis, and HlgA/BFB1 (residues 1–97) was unable to lyse erythrocytes at the doses tested (Fig. 3*B*).

We next tested binding of the chimeras to erythrocytes *via* flow cytometry and immunoblotting. These studies revealed that HlgBFB1 is severely attenuated in binding (Fig. 3, *C* and *D*). HlgBFB4 is also attenuated in binding, while HlgBFB2 appears to bind slightly better than WT (Fig. 3, *C* and *D*). HlgBFB2’s attenuation in hemolysis but not erythrocyte binding is likely due to domain 2 containing the stem domain, the region responsible for folding down into the host cell membrane for pore formation (44). It is possible that this chimera binds erythrocytes too tightly and makes subsequent stem domain unfolding more difficult.

Alternatively, HlgBFB1, HlgBFB2, and HlgBFB4 could have structural abnormalities inhibiting dimerization and/or pore formation, leading to their attenuated hemolysis. To explore this further, we performed cytotoxicity assays using hPMNs. While HlgA plus HlgBFB1, HlgBFB2, and HlgBFB4 showed a slight reduction in hPMN lysis, the decrease was minimal (Fig. 3*E*). Taken together, these data suggest that the observed attenuation in the chimeric proteins is specific to erythrocyte lysis and not due to structural abnormalities.

### Defining the role of the rim domain in HlgAB-mediated hemolysis

**Figure 4:**
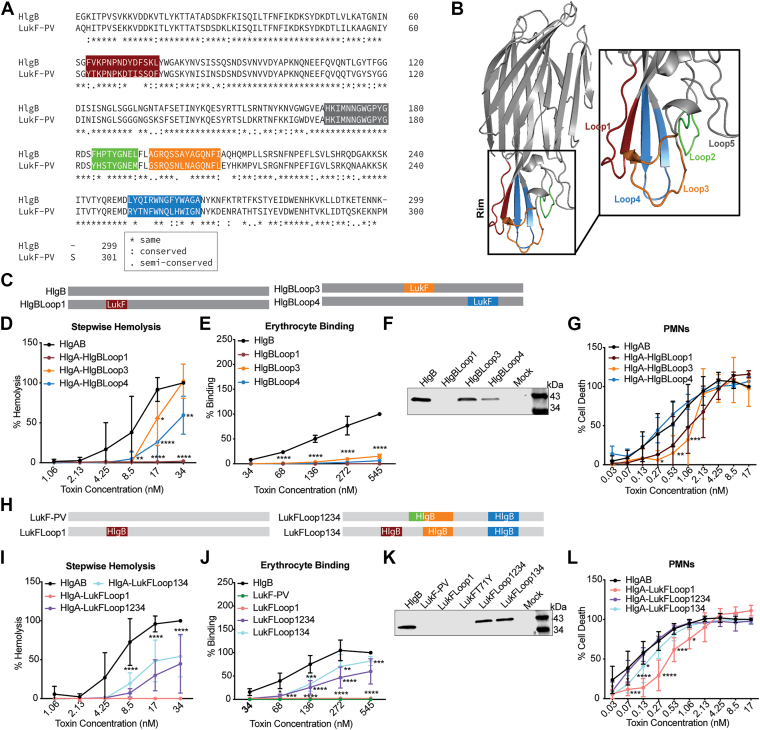
**HlgB loop 1 within domain 1 is necessary but not sufficient for erythrocyte binding by HlgB and lysis by HlgAB.***A*, ClustalOmega alignment of amino acid sequences for HlgB and LukF-PV with colors signifying divergent loops 1 to 5. *B*, atomic resolution structure for HlgB (1LKF), with 5 loops indicated. *C*, Diagram of the different HlgB constructs used in *panels* D-G. *D*, Stepwise hemolysis of primary human erythrocytes treated with F-subunit (WT HlgB or HlgB chimera), washed, and then treated with HlgA. HlgBLoop1 indicates HlgB with Loop 1 from LukF-PV, HlgBLoop3 indicates HlgB with Loop 3 from LukF-PV, *etc.* 34 nM of WT HlgAB was set to 100% (n = 4–8). *E*, binding of HlgB or HlgB chimeras to primary human erythrocytes as measured by flow cytometry. Data were normalized to 545 nM HlgB binding at 100% (n = 4). *F*, binding of WT HlgB or HlgB chimeras to primary human erythrocytes, measured by immunoblot with anti-His primary antibody. Immunoblot depicts a representative sample. *G*, viability of primary human PMNs treated with HlgA combined with WT HlgB or the HlgB chimeras. 17 nM of WT HlgAB was set to 100% (n = 4–12). *H*, diagram of the different LukF-PV constructs used in panels I-L. *I*, Stepwise hemolysis of primary human erythrocytes treated with F-subunit (WT HlgB or LukF-PV chimera), washed, then treated with HlgA. LukFLoop1 indicates LukF-PV with Loop 1 from HlgB. 34 nM HlgAB was set to 100% (n = 5–9). *J*, binding of HlgB, LukF-PV, or LukF-PV chimeras to primary human erythrocytes as measured by flow cytometry. Data were normalized to 545 nM HlgB binding at 100% (n = 4). *K*, binding of WT HlgB, LukF-PV, or LukF-PV chimeras to primary human erythrocytes, measured by immunoblot with Anti-His primary antibody. Immunoblot depicts a representative sample. *L*, viability of primary human PMNs treated with HlgA combined with WT HlgB or the LukF-PV chimeras. 17 nM of WT HlgAB was set to 100% (n = 4–8). The other data are pooled from at least two independent experiments and represent means ± SD. ∗∗∗∗, *p* < 0.0001; ∗∗∗, *p* < 0.001; ∗∗, *p* < 0.01 ∗, *p* < 0.05) two-way ANOVA with Sidak’s correction for multiple comparisons.

To narrow down the critical residues involved in erythrocyte targeting from the domains described above, we further focused on the rim domain, which has been implicated in host receptor recognition by other leukocidin subunits (14, 23, 24, 25, 26). There are 5 loops in the rim domain of HlgB, one of which is conserved between HlgB and LukF-PV (Fig. 4, *A* and *B*). Loop 1 is found within domain 1 (HlgBFB1), loop 2 and 3 in domain 3 (HlgBFB3), and loop 4 in domain 4 (HlgBFB4) (Fig. 4, *A* and *B*). We created new chimeras by swapping out these loops from LukF-PV into HlgB one at a time and tested these proteins in the stepwise hemolysis assay as before. HlgA/HlgBLoop3 (residues 195–208) showed similar levels of hemolysis to WT HlgAB, HlgA/HlgBLoop4 (residues 251–264) showed intermediate hemolytic activity, and HlgA/HlgBLoop1 (residues 63–76) was unable to lyse erythrocytes at the doses tested (Fig. 4*C*). We were unable to produce HlgBLoop2 (residues 184–192). The reduced hemolysis by HlgBLoop3 and HlgBLoop4 was accompanied with notable inhibition of binding to erythrocytes (Fig. 4, *D* and *F*). HlgBLoop1 exhibited no detectable binding to erythrocytes consistent with its lack of hemolysis (Fig. 4, *D* and *F*).

To determine whether the altered hemolysis and binding characteristics of the chimeras were cell type specific, and to once again check for issues with dimerization and pore formation, we next intoxicated hPMNs. HlgA/HlgBLoop4 showed similar levels of hPMN lysis as WT HlgAB, while HlgA/HlgBLoop1 and HlgA/HlgBLoop3 showed a slight attenuation in hPMN lysis (Fig. 4*E*). Thus, the severe attenuation in hemolysis and binding to erythrocytes observed with HlgBLoop1 is cell type specific.

Since loops 1, 3, and 4 seem to be important domains within HlgB that dictate binding and lysis of erythrocytes, we next created LukF-PV chimeras: with HlgB loop1 (residues 63–76; LukFLoop1), with HlgB loops 1, 3, and 4 (residues 63–76, 184–192, 195–208, 251–264; LukFLoop134), and with the entire HlgB rim domain (loops 1–4; residues 63–76, 195–208, 251–264; LukFLoop1234). LukFLoop1 did not bind or lyse erythrocytes but still lysed hPMNs (Fig. 4, *G*–*J*). LukFLoop1234 and LukFLoop134 had intermediate hemolytic and binding ability and lysed hPMNs like HlgAB (Fig. 4, *G*–*J*). LukF1234 did not perform better than LukF134, so loop 2 is unlikely to be directly involved in erythrocyte binding and lysis. Altogether, HlgB loops 1, 3, and 4 are important for erythrocyte binding and lysis, and loop 1 is necessary but not sufficient to enable LukF-PV to gain erythrocyte binding and hemolytic ability.

### Y71 in loop 1.2 of HlgB is required for erythrocyte targeting

**Figure 5:**
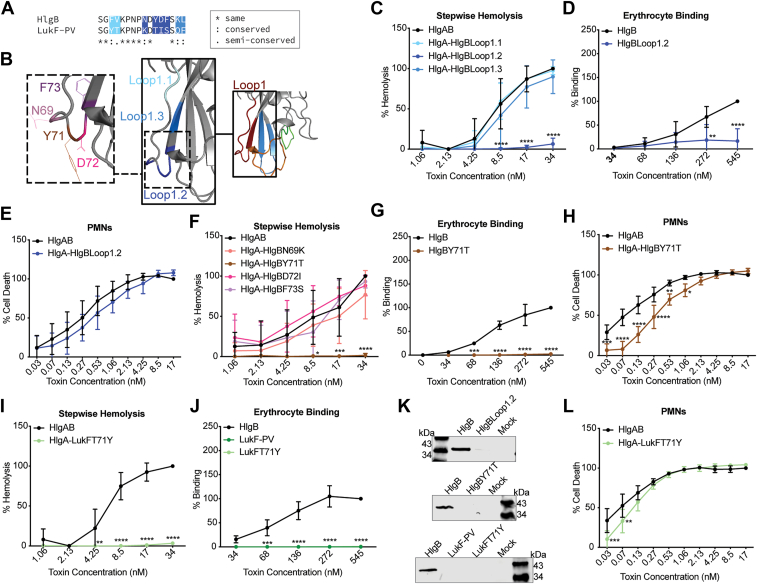
**Tyrosine 71 within loop 1.2 is necessary for erythrocyte binding by HlgB and lysis by HlgAB.***A*, ClustalOmega alignment of amino acid sequences for HlgB and LukF-PV with colors signifying divergent sections of loop1. *B*, atomic resolution structure for HlgB (1LKF), with 3 regions (all within loop 1) indicated. Four divergent residues of HlgB are labeled within loop1.2 within loop1. *C*, stepwise hemolysis of primary human erythrocytes treated with F-subunit (WT HlgB or chimera), washed, then treated with HlgA. BLoop1.1 indicates HlgB with loop 1.1 from LukF-PV, *etc.* 34 nM of WT HlgAB was set at 100% (n = 4). *D*, binding of HlgB or HlgB chimera to primary human erythrocytes as measured by flow cytometry. Data were normalized to 545 nM HlgB binding at 100% (n = 4). *E*, viability of primary human PMNs treated with WT HlgA and HlgB or HlgB chimera. 17 nM of HlgAB was set at 100% (n = 4). *F*, stepwise hemolysis of primary human erythrocytes treated with F-subunit (WT HlgB or chimera), washed, then treated with HlgA. HlgBN69K indicates HlgB with lysine (K) instead of asparagine (N), *etc.* 34 nM of WT HlgAB was set to 100% (n = 5). *G*, binding of HlgB or HlgB chimera to primary human erythrocytes as measured by flow cytometry. Data were normalized to 545 nM WT HlgB binding at 100% (n = 4). *H*, viability of primary human PMNs treated with WT HlgA and HlgB or HlgB chimera. 17 nM of WT HlgAB was set at 100% (n = 4). *I*, stepwise hemolysis of primary human erythrocytes treated with F-subunit (WT HlgB or LukF-PV chimera), washed, then treated with HlgA. LukFT71Y indicates LukF-PV with threonine (T) instead of tyrosine (Y). 34 nM of WT HlgAB was set to 100% (n = 5). *J*, binding of HlgB, LukF-PV, or LukF-PV chimera to primary human erythrocytes as measured by flow cytometry. Data were normalized to 545 nM WT HlgB binding at 100% (n = 4). Of note, LukF-PV and LukFT71Y are superimposed in the graph due to lack of binding. *K*, binding of WT HlgB or HlgB chimeras to primary human erythrocytes, measured by immunoblot with anti-His primary antibody. Immunoblots depict a representative sample. *L*, viability of primary human PMNs treated with WT HlgA and HlgB or LukF-PV chimera. 17 nM of WT HlgAB was set at 100% (n = 4). The other data are pooled from at least two independent experiments and represent means ± SD. ∗∗∗∗, *p* < 0.0001; ∗∗∗, *p* < 0.001; ∗∗, *p* < 0.01 ∗, *p* < 0.05; C-K) two-way ANOVA with Sidak’s correction for multiple comparisons.

We decided to focus on loop 1, as this loop had the greatest impact on HlgB-mediated erythrocyte binding and lysis. Within loop 1, there are 8 residue differences between HlgB and LukF-PV, which we split into three new groups (Fig. 5, *A* and *B*). Once again, we created chimeras by swapping analogous regions of LukF-PV into HlgB. HlgA/HlgBLoop1.1 (residues 63 and 64) and HlgA/HlgBLoop1.3 (residues 75 and 76) performed similarly to WT HlgAB in stepwise hemolysis, but HlgA/HlgBLoop1.2 (residues 69, 71–73) was highly attenuated (Fig. 5*C*). HlgBLoop1.2 exhibited negligible binding to erythrocytes, but when combined with HlgA, HlgBLoop1.2 was still able to lyse hPMNs like WT HlgAB (Fig. 5, *D*, *E*, and *L*).

Within loop 1.2, there are 4 residue divergences in HlgB and LukF-PV. We created new chimeras by swapping residues one at a time in HlgB to the amino acid in LukF-PV (Fig. 5*B*). HlgA/HlgBN69K, HlgA/HlgBD72I, and HlgA/HlgBF73S all exhibited similar levels of stepwise hemolysis to WT HlgAB, while HlgA/HlgBY71T was completely attenuated (Fig. 5*F*). HlgBY71T was also attenuated in erythrocyte binding (Fig. 5, *G* and *L*). In contrast, HlgA/HlgBY71T retained high cytotoxic activity toward hPMN (Fig. 5*H*). Thus, Y71 is required for HlgB targeting of human erythrocytes.

We then created a LukF-PV protein with the opposite amino acid swap (T71Y). LukFT71Y was unable to bind and lyse erythrocytes (Fig. 5, *I*, *J* and *L*), phenocopying the results with LukFLoop1. This LukF-PV chimera was still able to lyse hPMNs (Fig. 5*K*). Thus, while the tyrosine at position 71 in HlgB is critical for erythrocyte lysis, it is not sufficient to render HlgA/LukF-PV hemolytic.

### Assessing the importance of HlgB loop 1 in *S. aureus*-mediated hemolysis and pathogenesis

**Figure 6:**
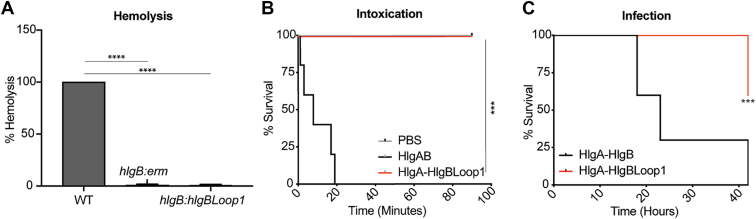
**HlgB loop 1 is required for *S. aureus* hemolysis and pathogenesis.***A*, hemolysis of primary human erythrocytes incubated with 5 × 10^6^ CFU/ml of the indicated *S. aureus* strains for 17 h (n = 3). Hemolysis induced by WT *S. aureus* was set to 100%. *B*, survival curve of mice challenged IV with purified WT HlgAB or HlgA/HlgBLoop1 chimera (n = 5 mice/group). *C*, systemic infection model. Mice were infected retro-orbitally with 2.5 x 10^8^ CFU of NewmanΔΔΔΔ strains containing pOS1-P*lukAB*-*lukAss-HlgA-His6* and NewmanΔΔΔΔ strains containing pOS1-P*lukAB*-*lukAss-His6* plasmids with indicated leukocidin genes (n = 10 mice/group). Mice were monitored twice daily for signs of mortality. The data are pooled from at least two independent experiments (except B) and represent means ± SD. ∗∗∗∗, *p* < 0.0001; ∗∗∗, *p* < 0.001; ∗∗, *p* < 0.01 ∗, *p* < 0.05; *A*) unpaired *t* test; *B*, *C*) Mantel-Cox test.

The studies described above demonstrated a critical role of HlgB loop 1 in hemolysis when purified toxins are employed. To further evaluate the importance of this domain in *S. aureus*-mediated hemolysis, we replaced the *hlgB* gene with a chimeric gene encoding for HlgBLoop1 into the chromosome of *S. aureus (hlgB::hlgBLoop1)*. Next, we incubated *S. aureus* WT, an *hlgB* deficient strain (*hlgB::erm*), and our *hlgB::hlgBLoop1* strain with primary human erythrocytes and measured hemolysis. *S. aureus* lysed erythrocytes in this tissue culture hemolysis model, a phenotype dependent on HlgB and HlgBLoop1 (Fig. 6*A*).

HlgAB targets erythrocytes, leukocytes, and endothelial cells. While we have examined the role of HlgB loop 1 in hemolysis and hPMN targeting, the role in endothelial targeting is unknown. To address this, we took advantage of the HlgAB murine intoxication model (12, 23). As reported previously, mice intoxicated with WT HlgAB intravenously declined rapidly and succumbed to the toxin challenge (Figs. 1*D* and 6*B*). In contrast, mice intoxicated with HlgA/HlgBLoop1 did not exhibit any signs of morbidity and survived (Fig. 6*B*).

We next examined the role of HlgBLoop1 during systemic infection. Mice were challenged with a *S. aureus* strain lacking all the leukocidins (NewmanΔΔΔΔΔ) containing plasmids that produce HlgA and HlgB or HlgA and HlgBLoop1. These studies revealed a noticeable reduction in murine mortality when mice were infected with strains expressing HlgA/HlgBLoop1 compared to HlgAB (Fig. 6*C*). Altogether, these data demonstrate that HlgB-mediated host targeting is important for *S. aureus* pathogenesis *in vivo*.

## Discussion

The ability of *S. aureus* to lyse erythrocytes to liberate hemoglobin, the richest source of nutrient iron in mammals, has been known for over a century. Moreover, HlgAB-mediated hemolytic activity has been noted since 1938, when Smith & Price purified the toxins (33). Despite the importance of leukocidin-mediated cell lysis in *S. aureus* infection, it is clear that the previously accepted model of toxin-mediated host targeting is incomplete. Indeed, erythrocyte targeting by not only S-subunit HlgA but also F-subunit HlgB is essential for HlgAB-mediated lysis (20, 30, 31, 32). Here, we combined detailed structure-function analyses together with hemolysis, cytotoxicity, cell binding, and *in vivo* studies to define the role of HlgB-mediated cell targeting in *S. aureus* pathobiology.

We confirmed that HlgB binds erythrocytes independent of its S-subunit counterpart, HlgA, and established that binding to erythrocytes is also independent of DARC, the HlgA receptor on erythrocytes. The DARC-independent binding to erythrocytes presented here is at odds with a recent publication by Grison *et al.* that shows HlgB binding to purified DARC in solution (45). We speculate that the difference in our findings is likely the result of the methods used by the authors, detergent-embedded DARC, and cells over-expressing DARC, where toxin binding might not reflect the physiological interactions that occur with the natural levels of the receptor embedded within the natural erythrocyte membrane. Alternatively, the HlgB-DARC interaction could be weak and only detected by the sensitive methods deployed by Grison *et al.* Regardless of whether HlgB binds DARC, it is clear that HlgB can bind primary human erythrocytes lacking DARC. The F-subunits have been shown to bind phosphatidyl-choline, but this does not explain how HlgB binds erythrocytes while LukF-PV does not (46, 47). Recently, HlgB was found to bind the autocrine motility factor receptor (AMFR), an ER-resident E3 ubiquitin ligase, during *S. aureus* survival intracellularly (48). As AMFR is ER-membrane anchored, a major question remains: What is HlgB binding on the surface of erythrocytes and/or endothelial cells?

Additionally, our stepwise intoxication of erythrocytes shows that adding HlgB before HlgA results in a much higher level of hemolysis. Because the S-subunit has been the predicted driver of specificity due to interactions with host cell receptors, the assumption is that this is the first step in leukocidin-mediated lysis. However, the data presented here, and that of others (31), suggest that for erythrocytes, HlgB interaction promotes HlgA recruitment and subsequent pore formation. Investigating each step in the pore-formation process on different target cells is an important avenue for future research.

To unearth the molecular details of how HlgAB interacts with erythrocytes, we created chimeras by taking advantage of the amino acid sequence similarity between HlgB and LukF-PV, the latter of which is unable to bind or lyse erythrocytes. Our studies revealed that several loops within the rim domain are critical for HlgB binding and HlgAB hemolysis. We showed that amongst the most debilitating alterations of HlgB is the mutation of tyrosine 71 (Y71) in loop 1 of the rim domain. This position is a threonine in LukF-PV and the difference in polarity of these two amino acids may explain our data. In addition, tyrosine and threonine are differentially phosphorylated, which could further affect interactions between this residue and proteins, lipids, or glycans (49, 50). Of note, a comprehensive literature search while writing this manuscript revealed a prior study by Yokota and Kamio that described the importance of Y71 in hemolysis and erythrocyte binding by HlgB (31). In the Yokota and Kamio study, HlgB was called LukF, which eluded our initial literature search (31). Our results differed slightly to that of the Yokota and Kamio study as they reported ∼20% hemolysis and ∼35% binding with both their HlgBY71T and LukFT71Y chimeras at 12 nM, while we measured <5% hemolysis and binding with these chimeras at our lowest concentration tested (34 nM), even after replicating their normalization process (31). Regardless, we confirmed the importance of tyrosine 71 in erythrocyte binding and lysis, while also showing that this residue is not sufficient. We further expanded upon this finding by demonstrating that this residue is not necessary for HlgAB-mediated hPMN lysis. Y71 is within a domain of HlgB we named loop 1, and this loop was found to be necessary but not sufficient for erythrocyte targeting. Loops 1, 3, and 4, however, conferred intermediate erythrocyte binding and lysis to LukF-PV, further demonstrating the role of the HlgB rim domain in this process.

The γ-hemolysin locus is present in >99% of sequenced human *S. aureus* isolates, as it is part of the core genome. *hlgA* has its own promoter, followed by *hlgC* and *hlgB*, which are cotranscribed from a different promoter and form the *hlgCB* operon (41). We performed a comparative genomic analysis of *hlgA* and *hlgB* and found both to be highly conserved across the *S. aureus* population. The near ubiquitous presence and minimal genetic diversity of these genes imply the importance of HlgACB in *S. aureus* pathogenesis. To this point, HlgBLoop1 (residues 63–76) was attenuated in the murine toxin challenge and systemic infection model. Because murine lethality by HlgAB intoxication is due to endothelial lysis (12), it is tempting to speculate that the HlgB chimera is also attenuated in targeting endothelial cells *in vivo*. The precise role of HlgB in HlgAB-mediated endothelial cell lysis and subsequent damage of vascular integrity is an area of current research in our lab.

In addition to HlgAB, LukED also lyses primary human erythrocytes. The mechanism of HlgAB-mediated and LukED-mediated hemolysis is likely similar, though it has been shown that LukED and HlgAB interact differently with DARC (13, 26). Like HlgB, LukD also binds erythrocytes independently of an S-subunit counterpart (20, 23). An in-depth structure-function analysis of LukD domains and their role in LukED-mediated hemolysis is an important topic of future study.

In sum, *S. aureus* leukocidins target different cell types to contend with the various defense mechanisms employed by the host, to provide nutrients, and to promote dissemination (2, 5, 27). Herein we describe a molecular blueprint of the role of HlgB in overcoming nutritional immunity and promoting *S. aureus* pathogenesis. This information not only improves our understanding of how leukocidins target host cells, but it also provides us with potential toxoids that could be developed into much needed vaccine antigens to combat *S. aureus* infections.

## Experimental procedures

### Ethics statement

Buffy coats were obtained from consenting, deidentified, healthy donors *via* the New York Blood Center or the Gulf Coast Regional Blood Center. All animal experiments were approved by the Institutional Animal Care and Use Committee of New York University and St Jude Children’s Research Hospital.

### Protein design and purification

All chimeric proteins are listed in Table S3. Chimeric proteins were synthesized as gBlocks (Integrated DNA Technologies or Azenta Life Sciences) and cloned into the pOS1-P*lukAB*-*lukAss-His6* plasmid using BamHI and PstI restriction sites. The plasmids were then transformed into *E. coli* IM08B, screened, sequenced, and then electroporated into *S. aureus* NewmanΔΔΔΔ (Δ*lukED hla::ermC hlgACB::tet lukAB::spec*) as described (14, 23, 24, 51). Toxins were purified from *S. aureus* Newman supernatants as described before (14, 23, 24, 51). Briefly, *S. aureus* NewmanΔΔΔΔ strains containing toxin expression plasmids were grown for 5 h, 180 rpm, at 37 °C. Culture supernatants were harvested *via* centrifugation and filtration and His-tagged proteins were purified using His-Trap HP His Tag protein purification columns (Cytiva) *via* FPLC. Chimeric proteins were purified similarly, but with HisPur Ni-NTA Resin (Thermo Scientific). Briefly, filtered subculture supernatants were incubated with resin in TBS, 10 mM imidazole for 45 min at 4 °C. Resin was washed with TBS, 25 mM imidazole and proteins were eluted with TBS, 500 mM imidazole.

All proteins were dialyzed into TBS, 10% glycerol and stored at −80 °C. Proteins were quantified by measuring A280 with NanoDrop 2000 UV-Vis Spectrophometer (Thermo Scientific) and multiplying by the molecular weight and dividing by the extinction coefficient. All holotoxin concentrations are represented per subunit.

### Hemolysis assays

Human erythrocytes were isolated from buffy coats from the New York Blood Center. 3% dextran in saline (AdipoGen Physiological Saline [Sodium Chloride 0.9%] Endotoxin-free Sterile Solution) was used to sediment erythrocytes, which were then washed three times with 0.9% NaCl *via* centrifugation at 2000 rpm, 4 °C for 7 min 4 × 10^6^ cells were incubated with purified toxins in cell-culture treated, 96 well, V bottom plates. For hemolysis assays, cells and proteins were incubated for 30 min at 37 °C, 5% CO_2_. Plates were spun down, and 50 μl of supernatant was transferred to a new, clear, 96-well flat bottom plate. Extracellular hemoglobin was measured as an indication of cell lysis at Absorbance 405 nm using a PerkinElmer EnVision plate reader. Each condition was performed in technical duplicate, and the values were averaged. Hemolysis was normalized to Triton-X100 positive control (set at 100%).

For stepwise hemolysis, erythrocytes were sedimented and washed as described above. Purified protein subunits were incubated at indicated concentrations with 4 × 10^6^ cells in a total volume of 100 μl of 0.9% NaCl in cell-culture treated, 96 well, V-bottom plates for 15 min on ice. Cells were then washed three times with 100 μl 0.9% NaCl. Toxin subunits were incubated with cells again for 15 min, and supernatant was collected after centrifugation and transferred to a new plate as described above. Extracellular hemoglobin was measured as an indication of cell lysis at Absorbance 405 nm using the PerkinElmer EnVision plate reader. Stepwise hemolysis was normalized to HlgAB at 34 nM (set at 100%). Each condition was performed in technical duplicate, and the values were averaged.

### Erythrocyte-binding assays

For flow cytometry experiments, human erythrocytes were isolated from buffy coats from the New York Blood Center as described above. Purified protein subunits were incubated at indicated concentrations with 4 × 10^6^ cells in a total volume of 100 μl of 0.9% NaCl in cell-culture treated, 96 well, V-bottom plates for 15 min on ice. Cells were washed twice with 100 μl 0.9% NaCl, then incubated with 25 μl phycoerythrin (PE) anti-His Tag antibody (clone J095G46), diluted 1:100 in 0.8% NaCl, for 25 min, 4 °C in the dark. Cells were then washed one more time, fixed with 100 μl FACS fixing buffer (2% FBS, 0.05% sodium azide, 2% paraformaldehyde in PBS), and analyzed by flow cytometry (CytoFLEX S). Each condition was performed in technical duplicate, and the values from the two wells were averaged.

To test binding *via* immunoblot, erythrocytes were isolated and washed as described above. Purified toxin subunits were incubated at 544 nM with 1.2 × 10^7^ cells in a total volume of 300ul of 0.9% NaCl for 15 min on ice. Cells were washed 3 times and then resuspended in 15 μl 0.9% NaCl. 15 μl 2× SDS loading buffer (100 mM Tris-HCl, 139 mM SDS, 20% glycerol, 294 mM 2-mercaptoethanol, 25 mM EDTA, 0.595 mM bromophenol blue, pH 6.8) was added and samples were boiled for 10 min. Lysates were run on a 10% SDS-Page gel and transferred onto a nitrocellulose membrane. Membranes were blocked for 30 min in 5% dry powder milk in PBS, 0.1% Tween-20. Anti-His primary antibody (1:3000; Cell Sciences clone HIS.H8) was incubated with membranes in 5% milk in PBST for 1 h at room temperature, shaking. After washing 3 times with PBST, membranes were incubated with Goat anti-Mouse IgG (H + L) Cross-Adsorbed Secondary Antibody, Alexa Fluor-680 (1:25,000; Invitrogen clone A21057) for 1 h at room temperature, shaking. Membranes were washed 3 more times with PBST, visualized by enhanced chemiluminescence, and imaged on Liacore.

### Measuring DARC expression

DARC expression was analyzed by flow cytometry. Human erythrocytes were isolated from buffy coats from the New York Blood Center as described above. 1 × 10^6^ cells in a total volume of 25 μl of 0.9% NaCl were incubated with 10 μl PE-conjugated, anti-DARC antibody (R&D Systems; clone 358307) or isotype control (R&D Systems cat. IC003P) in cell-culture treated, 96 well, V-bottom plates for 15 min at room temperature. Cells were washed twice with 100 μl 0.9% NaCl, fixed with 100 μl FACS fixing buffer (2% FBS, 0.05% sodium azide, 2% paraformaldehyde in PBS), and analyzed by flow cytometry (CytoFLEX S). Each condition was performed in technical duplicate, and the histograms depict a representative sample.

### Human neutrophil cytotoxicity assays

Human polymorphonuclear neutrophils (hPMNs) were isolated from buffy coats from the New York Blood Center using Ficoll-Paque method described previously (52). 1 × 10^5^ hPMNs were incubated with purified toxins at indicated concentrations in a total volume of 100 μl of RPMI 1640 (no phenol red, Thermo Fisher), 0.1% human serum albumin (HSA, Fisher Scientific, cat. AAJ6678003), 10 mM Hepes in a 96-well, TC-treated, flat bottom plate for 1 h at 37 °C, 5% CO_2_. To measure cell viability, 10 μl CellTiter (CellTiter 96 AQeuous One Solution Cell Proliferation Assay, MTS) was added to each well and incubated for 75 min at 37 °C, 5% CO_2_. Absorbance at 490 nm was measured using the PerkinElmer EnVision plate reader.

### Protein sequence alignments

The HlgB (from strain Newman) and LukF-PV (strain FPR3757) amino acid sequences were aligned with ClustalOmega 1.2.4 Multiple Sequence Alignment. The structural depictions of HlgB (PBD 1LKF) and LukF-PV (PDB 1PVL) were created using PyMOL 3.1.0.

### Comparative genomics

We extracted the full nucleotide sequences of the *hlgA* and *hlgB* genes from draft genome assemblies obtained from GenBank using an *in silico* PCR approach (https://github.com/egonozer/in_silico_pcr). The locus tags of *hlgA* and *hlgB* genes in the USA300 reference genome (GenBank accession: CP000255.1) are SAUSA300_2365 and SAUSA300_2367, respectively. We designed the 30 bp *in silico* PCR primers at the 3′ and 5′ ends of the gene sequences to generate amplicons spanning the full length of the gene from the start and stop codons. This ensured that the entire gene would be extracted even in the presence of a large insertion, deletion, or rearrangement in the internal fragment of the gene sequence. To ensure robust generation of the amplicons even in the presence of mutations, in the primer sequence at the ends of the genes, we allowed for one mutation in the primer sequences. After performing the *in silico* PCR and extracting the full gene sequences, we then selected the generated amplicons whose nucleotide sequence length was close enough to the length of the reference *hlgA* and *hlgB* to ensure there was no erroneous off-target amplification. We next translated the nucleotide sequences of each gene into amino acid sequences using BioPython (53).

We then defined specific alleles of the HlgA and HlgB genes as unique amino acid sequences. We calculated the frequency of each specific allele as the proportion of the amino acid sequences out of all the amino acid sequences. To assess the diversity of the HlgA and HlgB alleles, we calculated the Simpson diversity index using the vegan (version 2.6.8) (https://CRAN.R-project.org/package=vegan). Simpson diversity index close to zero indicates low diversity while values close to 1 shows high diversity. We also generated multiple sequence alignments of the representative allele sequences of the HlgA and HlgB proteins using MAFFT (version v7.505) (54). We then generated an unrooted maximum likelihood phylogenies of the representative HlgA and HlgB protein alleles based on the Le and Gascuel (LG) amino acid evolutionary model using PhyML (version 20151217) (55, 56). We processed and visualized phylogenetic trees of the HlgA and HlgB alleles using the “plot.phylo” function in the APE (version 5.8.1) package (57). We explored and generated visualizations of the alignments of the whole protein and only variable amino acid positions of the HlgA and HlgB protein alleles using SEAVIEW (version 5.0.5) (58).

We then generated a whole-genome phylogeny of the isolates to place the alleles in the context of the *S. aureus* population structure. We downloaded 2831 *S. aureus* genomes from the National Center for Biotechnology Information (NCBI) GenBank nucleotide database. We then generated a maximum likelihood phylogeny of the HlgA and HlgB proteins based on the LG model implemented in IQ-TREE (version 2.1.4-beta) (59). We then inferred the genetic clones or sequence types (ST) associated with each whole genome using the multi-locus sequence typing (MLST) approach using the scheme for *S. aureus* using mlst (version 2.23.0) (https://github.com/tseemann/mlst) (60). We visualized and annotated the whole-genome phylogeny using ggtree (version 3.12.0) (61). In addition, we plotted the association between the STs and specific alleles using a chord diagram generated using the “chordDiagram” function implemented in the rcircos (version 1.2.2) package (62).

### Murine toxin challenge

Six-week-old female Swiss Webster mice (Envigo) were intoxicated retro-orbitally in a final volume of 100 μl for 90 min. Time to acute intoxication was evaluated after sacrifice of mice displaying signs of morbidity, such as hunched posture, inability to walk, inability to consume food or water, or ruffled fur. All holotoxin concentrations are represented per subunit.

### Bacterial strains

WT bacterial strain is JE2. JE2 *hlgB::erm* was generated from the Nebraska library (USA300_JE2; SAUSA300_2367; clone NE1682) (63). JE2 *hlgB::hlgBLoop1* was created by cloning the gBlock of HlgBLoop1 into the site of *hlgB* on the chromosome using the pIMAY∗ (64). Briefly, Gibson assembly was performed with 2× Gibson master mix (Fisher Scientific), the gBlock (Integrated DNA Technologies), and the pIMAY∗ vector. The plasmid was then transformed into IMO8B and electroporated into JE2 *hlgB::erm* (clone NE1682). NewmanΔΔΔΔ (ΔlukED hla::ermC hlgACB::tet lukAB::spec) strains containing pOS1-P*lukAB*-*lukAss-His6* plasmids are listed in Table S5.

### *In vitro* hemolysis with *S. aureus* strains

NRPMI+ was created as described previously (13). *S. aureus* strains were grown overnight under iron-restricted conditions in RPMI (1640 -phenol red) supplemented with 1% Bacto casamino acids and 200 μM 2,2′-dipyridyl. Strains were sub-cultured 1:100 in 10 ml NRPMI+2,2′dipyridyl at 37 °C, 250 rpm until exponential phase. At OD ∼0.8, bacteria were spun down and washed twice with 10 ml chelex-treated NRPMI+ (Chelex-treated RPMI+ 1%CAS containing 25 μM ZnCl2, 25 μM MnCl2, 1 mM MgCl2, 100 μM CaCl2) supplemented with 500 μM 2,2′-dipyridyl and 10% Chelex-treated fetal bovine serum (FBS). Chelex treatment was performed with 7% (w/v) Chelex 100 shaking at 4 °C overnight. Erythrocytes were washed twice with NRPMI+ and resuspended to 5 × 10^8^ cells/ml. 50 μl diluted erythrocytes were combined with 50 μl diluted bacteria in a TC-treated 96 well V-bottom plate to a final concentration of 2.5 × 10^8^ cells/ml erythrocytes and 5 × 10^6^ CFU/ml bacteria. Bacteria and erythrocytes were incubated for 17 h at 37 °C. Plates were spun down, and 50 μl of supernatant was transferred to a new, clear, 96-well flat bottom plate. Extracellular hemoglobin was measured as an indication of cell lysis at A405 nm using the PerkinElmer EnVision plate reader. Each condition was performed in technical duplicate, and the values were averaged. Hemolysis was normalized to WT at 100% and media alone at 0%.

### Murine model of systemic infection

Eight-week-old female C57BL/6 mice were challenged retro-orbitally with 2.5 × 10^8^ CFU total of two strains: *S. aureus* NewmanΔΔΔΔ (Δ*lukED hla::ermC hlgACB::tet lukAB::spec*) containing the pOS1-P*lukAB*-*lukAss-HlgA-His6* plasmid and *S. aureus* NewmanΔΔΔΔ containing either pOS1-P*lukAB*-*lukAss-HlgB-His6* or pOS1-P*lukAB*-*lukAss-HlgBLoop1-His6* (100 μl inoculum). Animals were randomly assigned to infection groups. Before infection, mice were anesthetized intraperitoneally with 240 mg of Avertin (2,2,2-tribromoethanol dissolved in tert-amyl alcohol and diluted to a final concentration of 2.5% [vol/vol] in sterile saline) per kg of mouse. Mice were monitored twice daily for signs of mortality. Data is representative of two independent experiments.

### Statistics

Statistical significance was determined using Prism 9.0 (GraphPad Software). Two-way ANOVA with Sidak’s *post hoc* test for multiple comparisons was used to compare data sets with more than two strains. Unpaired t-tests were used for comparing data sets that only had two mutants. Log-rank (Mantel–Cox) test was used on survival data.

## Data availability

All data are contained within the manuscript.

## Supporting information

This article contains supporting information.

## Conflict of interest

The authors declare the following financial interests/personal relationships which may be considered as potential competing interests: V.J.T. is an inventor on patents and patent applications filed by New York University, which are currently under commercial license to Janssen Biotech Inc. Janssen Biotech Inc. provides research funding and other payments associated with the licensing agreement. All other authors declare no conflict of interest.
