# Supplementary material for: Cell targeting by the bicomponent leukocidin subunit HlgB drives Staphylococcus aureus pathophysiology
Source: J Biol Chem. 2025 Aug 12;301(10):110592. doi: 10.1016/j.jbc.2025.110592 (PMC12624776; doi:10.1016/j.jbc.2025.110592)
Supplement: Supplementary Material [file mmc1.docx]

**SUPPLEMENTARY MATERIALS**

**Supplementary Figure 1**
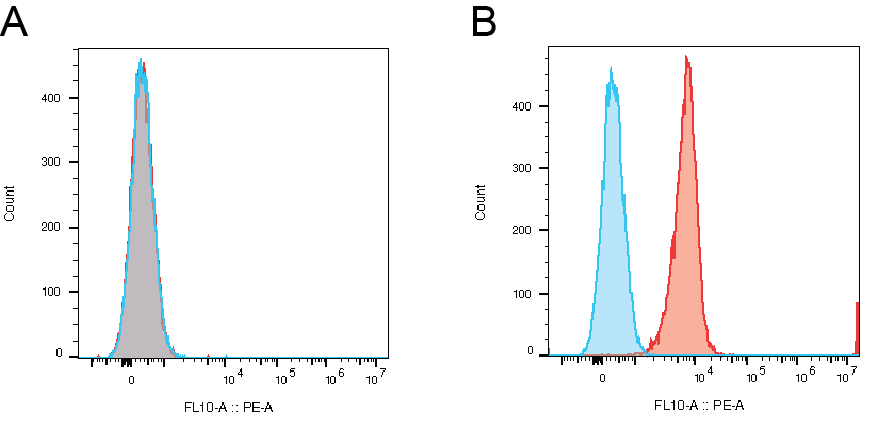


**Figure S1. DARC expression on DARC- vs DARC+ donor erythrocytes.** Level of expression of DARC on primary erythrocytes, as detected using DARC-specific monoclonal antibody (R&D Systems clone 358307). A) Red line indicates monoclonal antibody binding to cells. B) Isotype control monoclonal antibody (R&D Systems cat. IC003P) binding to both DARC+ and DARC- cells. Histograms depict a representative sample.

**Supplementary Figure 2**
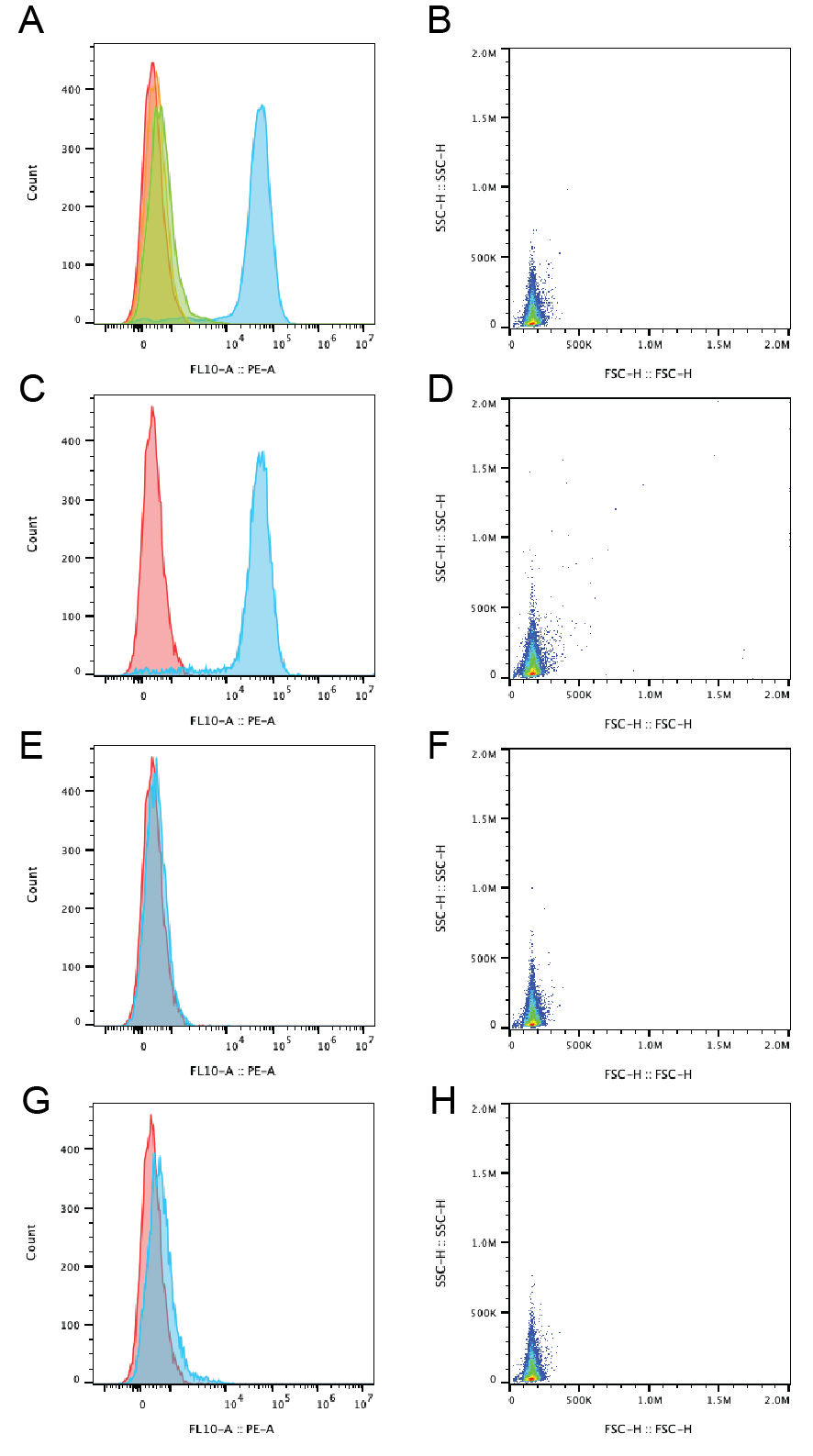


**Figure S2. HlgB, HlgBLoop1, or HlgBY71T binding to donor erythrocytes.** Binding of toxins or mock control to primary human erythrocytes, measured by flow cytometry with anti-His primary antibody. A) Blue line indicates HlgB bound to cells, red indicates mock control, orange indicates HlgBloop1, and green indicates HlgBY71T. B) FSC-H and SSC-H of mock treated cells. C) Blue line indicates HlgB treated cells, red indicates mock control. D) FSC-H and SSC-H of HlgB treated cells. E) Blue line indicates HlgBLoop1 treated cells, red indicates mock control. F) FSC-H and SSC-H of HlgBLoop1 treated cells. G) Blue line indicates HlgBY71T treated cells, red indicates mock control. H) FSC-H and SSC-H of HlgBY71T treated cells. These depict representative samples.

**Supplementary Figure 3**

**Figure S3. Conservation and population-level diversity of the HlgA and HlgB proteins**. Multiple sequence alignment of the HlgA and HlgB alleles showing only positions containing variable amino acids.

**Supplementary Figure 3**

**Figure S4. Conservation and population-level diversity of the HlgA and HlgB proteins**. Sequence alignment of the full amino acid sequences of the encoded *hlgA* and *hlgB* alleles.

**Supplementary Figure 5**


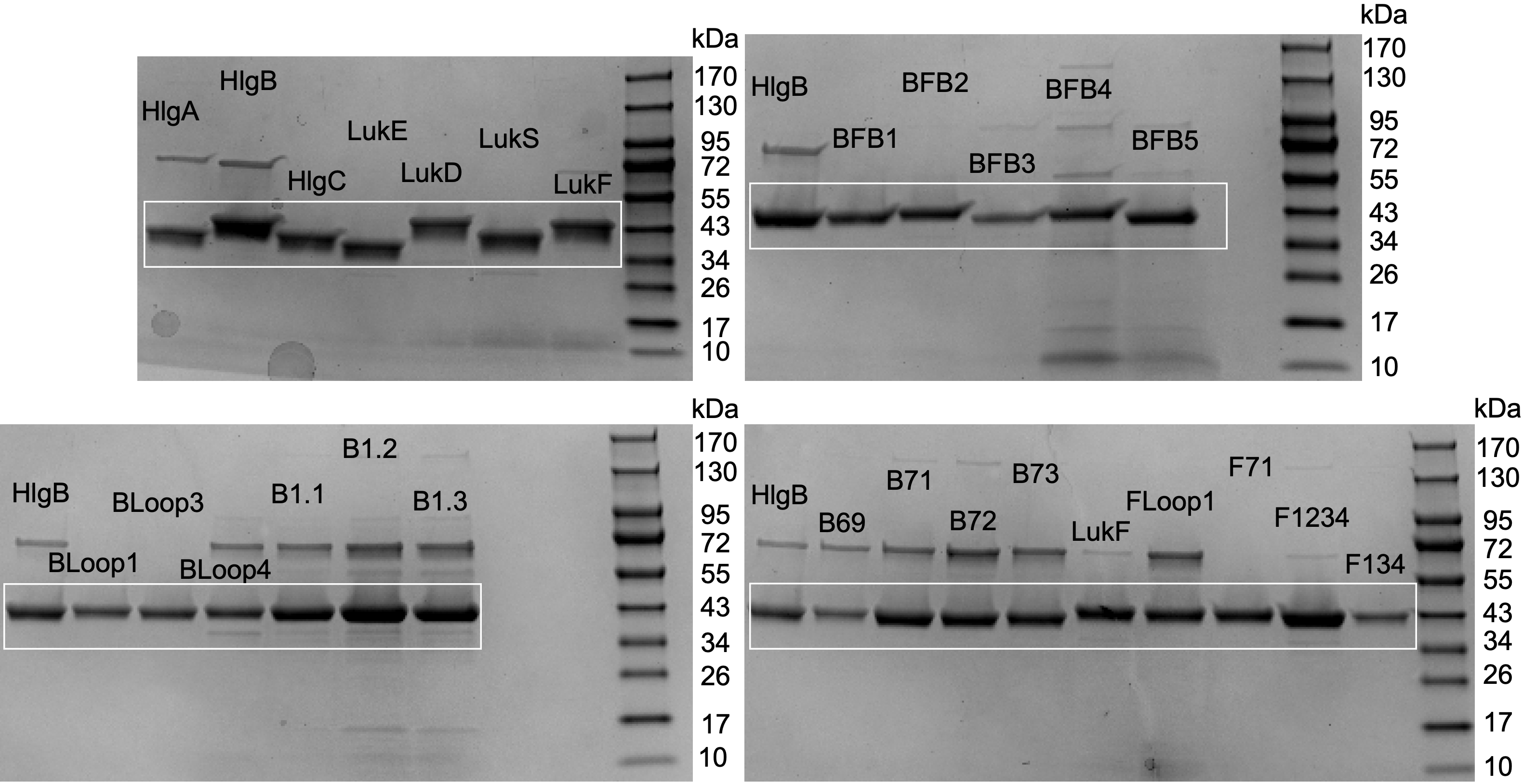
**Figure S5. Coomassie of all purified proteins.** Instant Blue stain of SDS-PAGE electrophoresis gel of all purified proteins, normalized to 1μg by NanoDrop. Leukocidin subunits are found within the white boxes.

**Supplementary Table 1. Summary of the HlgA alleles detected in the *Staphylococcus aureus* genomes used in the analysis**.

**Supplementary Table 2. Summary of the HlgB alleles detected in the *Staphylococcus aureus* genomes used in the analysis**.

**Supplementary Table 3. Chimeras used in this study.**

| **Chimera** | **Description** | **Residues** | **Depicted Color** | **Figure** |
| --- | --- | --- | --- | --- |
| HlgBFB1 | HlgB with domain 1 from LukF-PV | 1-97 | Pink | 3 |
| HlgBFB2 | HlgB with domain 2 from LukF-PV | 98-177 | Orange | 3 |
| HlgBFB3 | HlgB with domain 3 from LukF-PV | 178-222 | Green | 3 |
| HlgBFB4 | HlgB with domain 4 from LukF-PV | 223-267 | Blue | 3 |
| HlgBFB5 | HlgB with domain 5 from LukF-PV | 268-299/301 | Yellow | 3 |
| HlgBLoop1 | HlgB with loop 1 from LukF-PV | 63-76 | Maroon | 4 |
| HlgBLoop2 | HlgB with loop 2 from LukF-P; unable to produce | 184-192 | Green | 4 |
| HlgBLoop3 | HlgB with loop 3 from LukF-PV | 195-208 | Orange | 4 |
| HlgBLoop4 | HlgB with loop 4 from LukF-PV | 251-264 | Blue | 4 |
| LukFLoop1 | LukF-PV with loop 1 from HlgB | 63-76 | Pink | 4 |
| LukFLoop1234 | LukF-PV with loops 1, 2, 3, and 4 from HlgB | 63-76, 184-192, 195-208, 251-264 | Purple | 4 |
| LukFLoop134 | LukF-PV with loops 1, 3, and 4 from HlgB | 63-76, 195-208, 251-264 | Light blue | 4 |
| HlgBLoop1.1 | HlgB with loop 1.1 from LukF-PV | 63, 64 | Light blue | 5 |
| HlgBLoop1.2 | HlgB with loop 1.2 from LukF-PV | 69, 71-73 | Dark blue | 5 |
| HlgBLoop1.3 | HlgB with loop 1.3 from LukF-PV | 75, 76 | Medium blue | 5 |
| HlgBN69K | HlgB with lysine instead of asparagine at position 69 | 69 | Light pink | 5 |
| HlgBY71T | HlgB with threonine instead of tyrosine at position 71 | 71 | Brown | 5 |
| HlgBD72I | HlgB with isoleucine instead of aspartic acid at position 72 | 72 | Hot pink | 5 |
| HlgBF73S | HlgB with serine instead of phenylalanine at position 73 | 73 | Light purple | 5 |
| LukFT71Y | LukF-PV with tyrosine instead of threonine at position 71 | 71 | Lime green | 5 |

**Supplementary Table 4. Strains used in this study.**

| **Strain Name** | **Species** | **Description** | **Reference** |
| --- | --- | --- | --- |
| JE2 | *S. aureus* | Erm^S^ USA300 parent strain | Fey, et al. 2013.^62^ |
| JE2 *hlgb::erm* | *S. aureus* | JE2 containing erythromycin insertion into *hlgB* | Fey, et al. 2013.^62^ |
| JE2 *hlgb::bloop1* | *S. aureus* | JE2 containing *hlgBLoop1* insertion into *hlgB* | This study |
| NewmanΔΔΔΔ | *S. aureus* | Newman (Δ*lukED hla::ermC hlgACB::tet lukAB::spec*) | DuMont, et al. 2014.^64^ |
| NewmanΔΔΔΔ | *S. aureus* | Newman (Δ*lukED hla::ermC hlgACB::tet lukAB::spec*) and containing pOS1-P*lukAB*-*lukAss-His6* plasmid with recombinant leukocidin subunit. All iterations of this plasmid are listed below. | DuMont, et al. 2014.^64^ |

**Supplementary Table 5. Plasmids used in this study.**

| **Plasmids** | **Description** | **Resistance** | **Reference** |
| --- | --- | --- | --- |
| pOS1-P*lukAB*-*lukAss-His6*-HlgA | pOS1 expressing 6xHis-HlgA | Cm | DuMont, et al. 2014.^64^ |
| pOS1-P*lukAB*-*lukAss-His6*-HlgB | pOS1 expressing 6xHis-HlgB | Cm | DuMont, et al. 2014.^64^ |
| pOS1-P*lukAB*-*lukAss-His6*-LukS-PV | pOS1 expressing 6xHis-LukS-PV | Cm | DuMont, et al. 2014.^64^ |
| pOS1-P*lukAB*-*lukAss-His6*-LukF-PV | pOS1 expressing 6xHis-LukF-PV | Cm | DuMont, et al. 2014.^64^ |
| pOS1-P*lukAB*-*lukAss-His6*-HlgC | pOS1 expressing 6xHis-HlgC | Cm | DuMont, et al. 2014.^64^ |
| pOS1-P*lukAB*-*lukAss-His6*-LukE | pOS1 expressing 6xHis-LukE | Cm | DuMont, et al. 2014.^64^ |
| pOS1-P*lukAB*-*lukAss-His6*-LukD | pOS1 expressing 6xHis-LukD | Cm | DuMont, et al. 2014.^64^ |
| pOS1-P*lukAB*-*lukAss-His6*-HlgBFB1 | pOS1 expressing 6xHis-HlgBFB1 | Cm | This study |
| pOS1-P*lukAB*-*lukAss-His6*-HlgBFB2 | pOS1 expressing 6xHis-HlgBFB2 | Cm | This study |
| pOS1-P*lukAB*-*lukAss-His6*-HlgBFB3 | pOS1 expressing 6xHis-HlgBFB3 | Cm | This study |
| pOS1-P*lukAB*-*lukAss-His6*-HlgBFB4 | pOS1 expressing 6xHis-HlgBFB4 | Cm | This study |
| pOS1-P*lukAB*-*lukAss-His6*-HlgBFB5 | pOS1 expressing 6xHis-HlgBFB5 | Cm | This study |
| pOS1-P*lukAB*-*lukAss-His6*-HlgBLoop1 | pOS1 expressing 6xHis-HlgBLoop1 | Cm | This study |
| pOS1-P*lukAB*-*lukAss-His6*-HlgBLoop3 | pOS1 expressing 6xHis-HlgBLoop3 | Cm | This study |
| pOS1-P*lukAB*-*lukAss-His6*-HlgBLoop4 | pOS1 expressing 6xHis-HlgBLoop4 | Cm | This study |
| pOS1-P*lukAB*-*lukAss-His6*-HlgBLoop1.1 | pOS1 expressing 6xHis-HlgBLoop1.1 | Cm | This study |
| pOS1-P*lukAB*-*lukAss-His6*-HlgBLoop1.2 | pOS1 expressing 6xHis-HlgBLoop1.2 | Cm | This study |
| pOS1-P*lukAB*-*lukAss-His6*-HlgBLoop1.3 | pOS1 expressing 6xHis-HlgBLoop1.3 | Cm | This study |
| pOS1-P*lukAB*-*lukAss-His6*-HlgBN69K | pOS1 expressing 6xHis-HlgBN69K | Cm | This study |
| pOS1-P*lukAB*-*lukAss-His6*-HlgBY71T | pOS1 expressing 6xHis-HlgBY71T | Cm | This study |
| pOS1-P*lukAB*-*lukAss-His6*-HlgBD72I | pOS1 expressing 6xHis-HlgBD72I | Cm | This study |
| pOS1-P*lukAB*-*lukAss-His6*-HlgBF73S | pOS1 expressing 6xHis-HlgBF73S | Cm | This study |
| pOS1-P*lukAB*-*lukAss-His6*-LukFLoop1 | pOS1 expressing 6xHis-LukFLoop1 | Cm | This study |
| pOS1-P*lukAB*-*lukAss-His6*-LukFLoop1234 | pOS1 expressing 6xHis-LukFLoop1234 | Cm | This study |
| pOS1-P*lukAB*-*lukAss-His6*-LukFLoop134 | pOS1 expressing 6xHis-LukFLoop134 | Cm | This study |
| pOS1-P*lukAB*-*lukAss-His6*-LukFT71Y | pOS1 expressing 6xHis-LukFT71Y | Cm | This study |
| pIMAY* | Addgene 121441. Counterselector pheS*. | Cm | Shuster, et al. 2019^63^ |
| pIMAY* HlgBLoop1 | Addgene 121441 containing HlgBLoop1. Counterselector pheS*. | Cm | This study |

**Supplementary Table 6. Primers used in this study.**

| **Primer** | **Primer Sequence** |
| --- | --- |
| 1156 | 5’-CCCGGATCCGAAGGTAAAATAACACCAG-3’ |
| 3439 | 5’-CCCCTGCAGCTATTTATTGTTTTCAGTTTCT-3’ |
| 3478 | 5’-AAGCCTTAAAGACGATCCGGGAA-3’ |
| 3509 | 5’-ATTAGGCACCCCAGGCTTTACAC-3’ |
| 3630 | 5’-GTGGATCCCCCGGGCTGCAGTAAAACAAGTAATAAATGGG -3’ |
| 3631 | 5’-ATCTGCTGTGGCTGTTGTTTTGTATAAAGTAACTTTGTCATCGACTTTTT-3’ |
| 3632 | 5’-TACAAAACAACAGCCACAGCAGATTCTGATAAATTTAAAATTTCACAGAT-3’ |
| 3633 | 5’-CCCAGTAGAAGCCATTCCAACGAATTTGGTATAAATCCATTTCACGTTGA-3’ |
| 3634 | 5’-ATTCGTTGGAATGGCTTCTACTGGGCAGGCGCAAATTATAAAAAC-3’ |
| 3635 | 5’-GTACCGGGCCCCCCCTCGAGTAATGAAAGAGTGAC-3’ |
| 3638 | 5’-GCCCCCCCTCGAGCCTTAGCAATTGCTAGTTGTATCA-3’ |
| 3640 | 5’-TCGGTAAAGGAAGCGATATAGAAA-3’ |
| 3641 | 5’-ATTCTGTCCTTTCACCTTGATTTCA-3’ |
| 3642 | 5’-CAAAGGGGGATTAGGGCAAC-3’ |
